# Enabling Green and Blue Infrastructure to Improve Contributions to Human Well-Being and Equity in Urban Systems

**DOI:** 10.1093/biosci/biz058

**Published:** 2019-06-26

**Authors:** Erik Andersson, Johannes Langemeyer, Sara Borgström, Timon McPhearson, Dagmar Haase, Jakub Kronenberg, David N Barton, McKenna Davis, Sandra Naumann, Lina Röschel, Francesc Baró

**Affiliations:** 1 Stockholm Resilience Centre, at Stockholm University, in Stockholm, Sweden; 1a North-West University, in Potchefstroom, South Africa; 2 Institute of Environmental Science and Technology at the Universitat Autònoma de Barcelona, in Cerdanyola del Vallès, Spain; 3 Hospital del Mar Medical Research Institute, in Barcelona, Spain; 4 Department of Sustainable Development, Environmental Science and Engineering, at the Royal Institute of Technology—KTH, in Stockholm, Sweden; 5 Urban Systems Lab, The New School, in New York, New York; 6 Cary Institute of Ecosystem Studies, in Millbrook, New York; 7 Institute of Geography at Humboldt Universität zu Berlin, in Berlin, Germany; 8 Helmholtz Centre for Environmental Research—UFZ, Department of Computational Landscape Ecology, in Leipzig, Germany; 9 Faculty of Economics and Sociology at the University of Lodz, in Lodz, Poland; 10 Norwegian Institute for Nature Research, in Trondheim, Norway; 11 Ecologic Institute, in Berlin, Germany

**Keywords:** green and blue infrastructure, multifunctionality, urban social–ecological systems, environmental justice, resilience

## Abstract

The circumstances under which different ecosystem service benefits can be realized differ. The benefits tend to be coproduced and to be enabled by multiple interacting social, ecological, and technological factors, which is particularly evident in cities. As many cities are undergoing rapid change, these factors need to be better understood and accounted for, especially for those most in need of benefits. We propose a framework of three systemic filters that affect the flow of ecosystem service benefits: the interactions among green, blue, and built infrastructures; the regulatory power and governance of institutions; and people's individual and shared perceptions and values. We argue that more fully connecting green and blue infrastructure to its urban systems context and highlighting dynamic interactions among the three filters are key to understanding how and why ecosystem services have variable distribution, continuing inequities in who benefits, and the long-term resilience of the flows of benefits.

Nature contributes to human well-being in many different ways. Within science, as in other knowledge systems, there is a deep understanding of how to appraise and manage nature for different ecosystems. However, the often narrow and targeted approaches need to be placed in a larger context to better understand management options and their broad implications. In this article, we will use urban green and blue infrastructure (GBI) to demonstrate a systems approach to assess the conditions under which ecosystem services (ES) may be turned into various benefits for people and to critically ask questions about the distribution and resilience of the flow of diverse benefits.

Green and blue spaces and their functional connections and interrelations within and adjacent to cities have the potential to provide a broad range of ES to urban residents (e.g., Gómez-Baggethun et al. 2013, Haase et al. 2014). By addressing pressing issues such as temperature increases, poor environmental quality, and limited social inclusion, GBI is also held to contribute to the mitigation of broader urban sustainability challenges, such as climate change impacts, needs for outdoor recreation, and spaces for social activity (Kabisch et al. 2017, Elmqvist et al. 2018).

However, there are documented problems with the distribution and accessibility of both GBI and its benefits (*sensu* Fisher et al. 2009, Haines-Young and Potschin 2010). The uneven distribution of benefits has clear implications for when and for whom GBI offers an opportunity to meet different ES needs (Webster 2007, Reichl 2016, Haase et al. 2017). The failure to deliver ES equitably is currently discussed in terms of differences in biophysical landscape conditions and overall urban morphology; urban development pathways (Haase et al. 2017); institutional arrangements, such as property rights and governance schemes (Biernacka and Kronenberg 2019); current power regimes and procedural justice (Low 2013); and, closely related to the latter, historical legacies of social inequity and structural racism (e.g., Bullard 1993, Boone et al. 2009). However, our understanding of how factors external to GBI affect its overall functionality and contribution to the urban system it is embedded in is still incomplete. In a time when cities are undergoing rapid change—more rapid (and new modes of) and affordable transportation or flow of people or information, decreasing ecological connectivity within GBI but also new niches for urban species, shifts in public opinion and land tenure, new venues and processes for deliberation and decision-making (e.g., new public management), and value articulation are some examples—more conceptual work, as well as empirical work, on contextual factors is needed to improve the resiliency of the supply and to ensure an equitable distribution of benefits.

To improve our knowledge about how to better use GBI’s full potential, it is necessary to understand the translation of diverse ES into various benefits. The recent literature has increasingly stressed the role of coproduction of ES—that is, the importance of people and contextual circumstances (e.g., Ernstson 2013, Andersson et al. 2015, Palomo et al. 2016, Díaz et al. 2018). For different types of ES, this coproduction will look very different. For some regulating ES, such as air pollution removal and ambient temperature regulation, the key factor is the spatial configuration of the urban land cover mosaic at a neighborhood level (Hamstead et al. 2016) and the relations between supply and demand areas (Fisher et al. 2009, Syrbe and Walz 2012). For several cultural ES, such as aesthetics or beneficial nature experiences, it is primarily people's diverse individual preferences and shared (or conflicting) norms (Vatn 2005). Still, for other ES, such as active outdoor recreation and food production, it is land tenure and management (Kremen 2005, Langemeyer et al. 2015). Andersson and colleagues (2015) argued that this variation could be discussed relative to systemic factors such as institutions, available equipment, and technology. These factors do not provide ES themselves; instead, they either mediate or hinder the flow of benefits and therefore affect the distribution of benefits to a diverse set of potential beneficiaries. This context dependence is particularly prominent in cities in which the mutual dependence of nature and humans is both kaleidoscopic, because of the density of people and diversity of preferences and perspectives, and enmeshed in a human-dominated system of multiple layers of institutions and technical infrastructures (McPhearson et al. 2016).

Therefore, a more systemic and context- and situation-sensitive approach is needed to answer questions about who benefits from GBI and why and how we can govern critical components of the urban system to accommodate different needs and unequal opportunities. We propose that such an enabling approach to improve the resilient flow and equitable distribution of ES benefits should focus on three key factors: infrastructures, institutions, and perceptions. Each factor acts as a filter directly or interactively together with the other factors suppressing or enhancing the flows of different ES benefits.

## Enabling the flow of ES benefits

The ENABLE project (Enabling Green and Blue Infrastructure Potential in Complex Social–Ecological Regions, projectenable.eu) has developed a systems-based critical assessment and implementation approach for improving more equitable and resilient flow of ES benefits in urban contexts. The approach supports both designed mixed methods studies and *post hoc* expansion of narrowly framed research questions or management interventions. The ENABLE approach aims to advance the knowledge of how to work with the functionality of GBI more effectively and equitably—that is, how to negotiate trade-offs between different interests and interventions and to ensure the delivery of social and environmental benefits across diverse beneficiary groups over space and time, given continuous dynamic system change.

**Figure 1.**
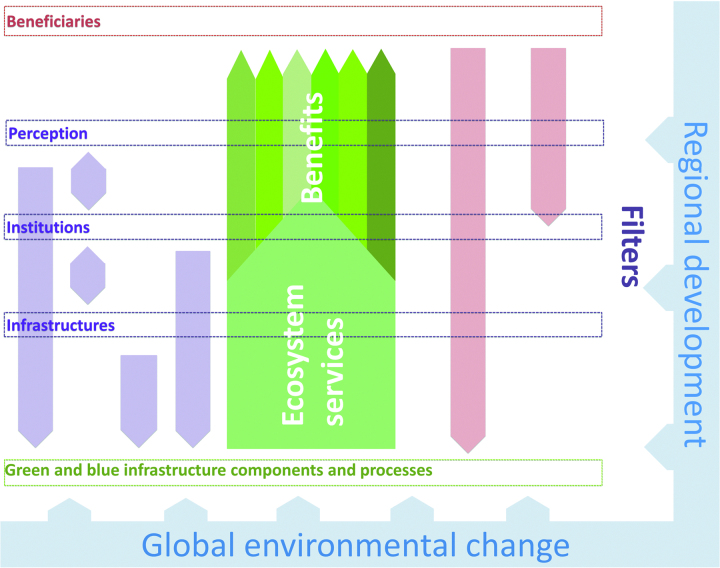
The systems model. The green and blue infrastructure components and their different ecological qualities provide the first necessary precondition for ecosystem services. Systemic factors (the purple boxes) can enable or disable the flow of ecosystem services and thus influence translation of ecosystem services into various benefits to beneficiaries (the red box). Downward oriented arrows represent the feedback from beneficiaries and other actors (the red arrows) and different filters (the two-way purple arrows) that can influence either other filters or the green and blue infrastructure components that underlie ecosystem service supply. These dynamics are then embedded in larger scale change exemplified in the article (but not necessarily restricted to) land-use change at a regional scale and environmental change at a global scale.

Our approach is focused on the identification of and analytical attention to the three aforementioned interconnected systemic factors important for the flow of benefits: the layout and intersection of infrastructures, including GBI and transportation networks and built (residential, commercial) areas; the institutional arrangement around GBI (i.e., factors such as ownership and user rights, policy intentions, and prescriptions), together with its implementation; the perceptions, understanding, and preferences of the beneficiaries, the numerous social and cultural factors that influence which benefits are ultimately available to people (figure 1). The next sections describe how these filters can be approached analytically and methodologically (table 1) and illustrate how they intersect with the flow and distribution of ES benefits.

**Table 1.** Capturing the necessary preconditions for ES benefit realization.

Filter	When is the filter particularly relevant?	Primary influence on flows of ES benefits	Descriptors and parameters	Methods
Infrastructure	When supply and demand do not coincide or where benefits are dependent on additional facilities or mediation	Functional connectivity between types of GBI and areas for housing, services and work. Physical barriers (for good and bad) and gray influence on the biophysical environment (externalities), creating need.	Quality, character and interconnections of infrastructures, network structure and topology, relations between source and demand, transportation options	Spatial analyses of urban morphology, environmental quality monitoring and modeling, mapping and modeling mobility options
Institutions	When benefits are strongly associated with either active use (of a resource) or a clear good (especially when the good is in limited supply). Institutions also have a second-order effect on benefit distribution or accessibility by regulating mobility.	Regulation of planning design and practical management and use. Articulation of values and goals or agenda setting. Restrictions to public access, formally or informally regulating activities that enable different benefits (e.g., recreation).	Land ownership and tenure rights, content of policy and access to policy formation processes including influence on value articulating institutions, actor mandates, social norms, management rules, policy alignment	Policy analysis by the use of documents, interviews and modeling. Participatory, multistakeholder assessments
Perceptions	When benefits are subjective, cocreated, intangible, relational or context dependent	Individual differences in how opportunities afforded by infrastructure and institutions are perceived and valued, relational dimensions of value, knowledge as an enabling factor	Demographics, socioeconomic status, value orientations, knowledge (available and held), learning	Interviews, questionnaires, behavioral or preference observation, modeling ­ (agent-based models)

## GBI connections to other urban infrastructures

City regions are defined by dense and predominantly built (grey) infrastructures that support major functions such as safety, transportation, communication, illumination, water supply, sanitation, and energy provision. The different infrastructures and their interconnections are primarily important for ES with a clear spatial dynamic (*sensu* Fisher et al. 2009)*.* Individual elements must be understood in terms of how they connect to elements from multiple different types of infrastructure (e.g., green roofs to green walls, transportation infrastructure connecting GBI to residential areas, or being greened themselves such as grass rails) and not just in terms of how they spatially and functionally connect to other elements of the same type (e.g., tree to tree or park to park; McPhearson et al. 2016). For example, transportation may cause environmental problems (e.g., anthropogenic contaminants in storm water, barriers to species’ movements, and emission of particles), creating a place-specific need for certain ES. In this case, the function of GBI is to prevent the spread of negative externalities across other infrastructures. Another example, relevant for a different group of ES, is when transportation is needed to make the recreational opportunities of a larger GBI component available to the residents of a non-neighboring housing area.

Where policy, planning, and management have historically treated grey and green infrastructural networks as separate, we now see a trend of increasing integration for using GBI to address urban resilience and sustainability goals together with prevailing technical infrastructures (Grimm et al. 2016, Kabisch et al. 2017, Meerow and Newell 2017). Examples of integration include hybrid solutions (e.g., urban runoff and sustainable drainage systems or heat protection installations; Depietri and McPhearson 2017) and integrated planning frameworks (e.g., Pauleit et al. 2019). Therefore, we discuss complementary grey, green, and blue infrastructural systems in cities—altering in the dominance of grey and either green or blue—as the extended infrastructure supporting and directing the flow of ES benefits.

The extent to which the combined infrastructures enable or obstruct functional links differs across groups and individuals. For example, differences in mobility (and in the ability to use different transportation infrastructure) are among the better recognized (and in the sense of access to GBI being physical sites, relatively easy to work with) causes of unequal access. Certain age groups or people with functional limitations may need special infrastructure or transportation options to reach a green space for recreation (Geurs and Van Wee 2004). In addition, more vulnerable groups (e.g., low socioeconomic status) tend to be more exposed to negative externalities or environmental burdens (e.g., Jerrett et al. 2001, Padilla et al. 2014).

The assessment of integrated infrastructures needs to combine quantification and evaluation of the potential of GBI (both its different elements and as a whole) to provide ES and to assess this information relative to areas of need or demand for the potential ES benefits. Depending on the benefit, the latter may include residential areas, transportation routes, and workplaces. Although physical distance offers a starting point, transportation networks and modes of transportation often offer a better approximation for how available GBI and ES benefits might be (e.g., Van Herzele and Wiedemann 2003). For example, a well-known green space with multiple connections to public transportation and developed recreational facilities, such as benches and picnic areas, might be more accessible than a nearby green space that is not connected to the transportation network and that lacks additional (quality of stay) facilities. Additional prerequisites for different benefits (e.g., location of a pollution source upwind or upstream relative to vulnerable areas, ambient noise, auxiliary facilities for recreation or transportation infrastructure) need to be mapped and used to condition the landscape potential. Weber and colleagues (2014) showed that the availability of green space in combination with the height of buildings helped reduce noise pollution in residential areas of cities. Larondelle and colleagues (2014) showed how water surfaces in the vicinity of built spaces lower the ambient air temperature more effectively than simply increasing the share of open space in a neighborhood. Together, these analyses represent a spatially explicit baseline map of both technical and green or blue infrastructures most relevant for the flow of ES benefits that can serve both at aggregated (whole city, urban region) and disaggregated (neighborhood, single trees) scales.

## The institutional setting: Land-use rights and collectively defined goals

Many benefits are realized through the active use of GBI (activities or activities in combination with extraction of goods, such as edible plants). Actor roles, rights, and responsibilities are framed by institutions, defined in this article as the formal and informal rules of a governance system. These, in turn, need to be understood as situated in a certain social, economic, and political context at a certain time (North 1991, Ostrom 2009). Together with the physical infrastructures of the urban landscape, the institutions provide a setting that individuals and groups can then use in different ways, pursuing different opportunities and benefits. Institutions articulate collective or shared values that reflect the individuals’ roles and perceptions and the norms they adhere to in their social contexts—for example, through political ambitions and priorities (Jacobs 1997, Vatn 2005). The institutional context, such as sectoral, jurisdictional, and administrative divisions, is often the basis for GBI management and use (Borgström et al. 2006). In addition to understanding how current policies influence the accessibility of benefits, we see institutions also as ongoing, often cyclic, processes. Civic movements, planning cycles, and continued policy revisions offer opportunities to influence the distribution of benefits and to reframe targets. Therefore, in our approach, we see institutions as also framing the ways people can be involved in changing how land is used.

Opportunities to realize different ideas and needs are not equally open to everyone (Biernacka and Kronenberg 2018, Czembrowski et al. 2019). A first sharp divider is the difference between private and public, and ownership history is a key determinant of present-day property rights, as well as the expectations of responsibility and access. Detailed land-use planning and property laws set the stage for formal and informal tenure arrangements and property rights, in turn affecting who can enjoy what benefits where and who has the opportunity to shape the development of GBI and the overall landscape (Biernacka and Kronenberg 2018, Langemeyer et al. 2018). For example, urban gardens, whether they are private or community owned, share many of the same potential benefits, but the groups of beneficiaries (and the terms of their involvement) differ (Colding and Barthel 2013). Higher socioeconomic status often affords opportunities to buy extended user or ownership rights and, therefore, access to both decision-making and direct management (de Magalhães and Freire Trigo 2017). Differences in the ability to be heard and influence policy processes manifest, for example, in gaps between individual preferences and the more consensus-oriented group values as expressed in policy objectives, sanctioned activities, and management plans (Ernstson 2013). This last points to the importance of openings for participating in value articulation and decision-making. For example, Colding and Barthel (2013) argued that diversity is needed in institutional arrangements in order to match different people's abilities and motives for participating, in turn influencing the overall inclusivity and opportunity for people to engage in the realization of desired benefits.

Similar to the physical infrastructure, a fragmented policy setting in which sectors are not aligned in terms of their targets, management strategies, or monitoring and evaluation (Borgström et al. 2006, Stead and Meijers 2009, Cejudo and Michel 2017) may reduce the contribution of GBI to human well-being. Information about the institutional setting needs to be added to the baseline maps of infrastructural systems by overlaying the physical landscape with information on land ownership, user rights, and formal and informal restrictions, stakeholders, and policy targets. At the local scale, this may include land-use zonation, special maintenance, and use contracts that promote a more narrow range of ES benefits than the combination of GBI type, ownership, and regulations generally would afford (e.g., by highlighting only a smaller selection of potential uses). However, an important source of injustice in access to GBI benefits can be found in the differences in whose values and preferences are captured or recognized by prevalent institutions. In addition to the spatial manifestation of different institutions, studies of the governance system can identify systemic opportunities and constraints for influencing and changing current conditions (e.g., Allen and Cochrane 2010, Silver et al. 2010).

## Individual perceptions, values, and experiences

Culture is an ever-present filter influencing how different individuals interpret different environments and circumstances (Stephenson 2008). Specific needs, knowledge, practices, identities, beliefs, worldviews, literature, and art all influence the planning, design, and practical management of the GBI sources of ES flow and which ES benefits are desired, realized, and recognized and in what form (Setten et al. 2012, Kenter 2016). Age, gender, ethnicity, and other cultural and socioeconomic circumstances may further accentuate these differences. For example, the opportunity to be physical active has been highlighted as an important benefit by elderly engaging in urban gardening (Langemeyer et al. 2018). Although younger people can be assumed to be as physically active while engaging in the same type of gardening activities, they do not place the same emphasis on this as an important benefit (Langemeyer et al. 2018). Moreover, the subjective perception is relevant not only for the individual appreciation of importance and value but also for the interpretation of the opportunities offered by GBI. Returning to the example of urban gardening, although women in Southern Europe are less likely than men to engage in gardening activities (Camps-Calvet et al. 2016), the opposite has been observed in Northern Europe (Barthel et al. 2010). The example indicates that the individual potential to realize ES benefits is closely interrelated and shaped by the cultural and institutional context.

Even when there are no formal restrictions, differences in knowledge, education, available information, and individual circumstances may privilege some voices and interests more than others (Schlosberg 2009, Sen 2009). The differences in opportunities to realize individual interests remain underdeveloped in ES research and practice (Ernstson 2013, Berbés-Blázquez et al. 2016). One of the foundations for enhancing equal opportunities to realize ES benefits in relation to multifunctional GBI is the understanding of the plurality of values and different needs and abilities among present and future beneficiaries and the extent to which they can realize these desires (e.g., Webster 2002, Agyeman 2013). Strategies for making spaces more multifunctional and more used often assume compatibility of use, but this is not always possible. The activities associated with realizing different benefits may be directly in conflict (e.g., Dinnie et al. 2013, Biernacka and Kronenberg 2018), or the value of a specific benefit may deteriorate if too many people seek to realize it at the same time (e.g., quiet and restorative experiences of nature). In addition, accessibility also has a psychological dimension, related to the perceptions of a given place based on unwritten social norms and the prospective users’ negative feelings (in particular, of not being welcome there or feeling unsafe). Therefore, increased accessibility needs to be viewed in the light of institutional conditioning and governance structures that can reduce or help resolve such trade-offs (Biernacka and Kronenberg 2018).

It is clear that individual perspectives on values and opportunities, as well as benefit interdependence, need to be taken into account in research and planning. These have, up until now, been relatively unexplored. The methods chosen for assessing the experiential quality of the urban landscape need to be sensitive to the nature of the benefit, the conditions under which it is available (e.g., Bergseng and Vatn 2009) and the interpersonal differences among beneficiaries (with regard to their ability to realize and recognize benefits). Assessing different benefits and their values requires tailored methods (e.g., Harrison et al. 2018). Depending on which component of GBI is being explored, the urban context it is set in, and the scale of study, the methodological design for capturing these plural values needs to be case and context specific. In turn, the context-specific nature of benefits means that extrapolating research findings even among neighborhoods in a single city requires great caution, if it can be done at all.

## Embedded multifunctionality and cities in constant change

Beyond the critical assessment of the current distribution of ES benefits, the second question the ENABLE approach can provide is a solid basis for addressing what lies ahead. Cities and the people in them are facing upcoming changes that will influence both the need for different ES and the distribution of benefits (exemplified in box 1). Some of these drivers of change are external, such as climate change or large-scale trade or migration patterns, whereas others are internal and connected to changes in the three filters. Already, changes such as increasingly hybridized infrastructures, increasing privatization of open public space (Lee and Webster 2006), and new worldviews and expectations are already evident in many cities (Pereira et al. 2018). With compact or smart cities being one of the current paradigms for urban growth, the addition of new GBI is often paralleled by the loss—or at least geographic displacement toward the urban periphery or the rooftop level—of other types of GBI (Westerink et al. 2013, Haaland and van den Bosch 2015). This shifts the baseline for where different ES might be available in the densified urban landscape (Lin and Fuller 2013). Moreover, more and more people will look to remaining GBI to satisfy their ES needs and desires (Gren et al. 2018). These different trends make resilience, in addition to justice, a key question for planning, governing, and managing GBI (e.g., McPhearson et al. 2016, Andersson et al. 2017). Increasing recognition of the many values tied to GBI is driving both an active engagement with the biophysical structure of GBI itself (e.g., by the rollout of new types of GBI such as green roofs across the urban landscape) and the larger system around it. The latter includes both interventions for increased accessibility (e.g., by increasing affordable transportation access, providing information and adding amenities to GBI) and measures for building resilience against different threats (e.g., extreme events but also vandalism). However, making GBI itself and the factors that enable flows of ES benefits resilient has received less attention (McPhearson et al. 2015, Andersson et al. 2017).

**Box 1. Systemic filters in action: The case of Barcelona.**

Barcelona is a compact Mediterranean city with little per capita urban green space relative to other European cities (Baró et al. 2014), especially in the city center. It is currently challenged by climate change and human health issues (e.g., heat waves, flash floods, and air pollution), placing the question of how to unlock or redirect flows of GBI benefits high on the agenda. The city is also undergoing a process of shifting user preferences and expectations in response to new value framings, new lifestyles, and demographic changes. Increasing use by both residents and visitors is creating new trade-offs and, with them, new institutional and management challenges. For example, recreational activities (e.g., cycling, trail running) have substantially increased in the periurban natural area of Collserola (currently protected as a natural park) during the last decade, putting pressure on biodiversity and other disturbance sensitive benefits.

As a first step toward finding workable solutions, the city council has approved several strategic policies in order to enhance and increase GBI within the municipality (e.g., Barcelona City Council 2018). New types of hybrid green–blue–grey infrastructure have opened up new options for where greening is possible (e.g., the creation of urban parks such as the Jardins de la Rambla de Sants, on top of railway infrastructures, and the recent municipal funding of 10 green roof projects). This makes it possible to target and repurpose problematic or underserved areas in which traditional GBI elements, such as parks or gardens, are impossible. Eventually, new types of GBI and mobility options are intended to provide a more equitable distribution of GBI benefits, especially in terms of cultural ES and regulating ES such as runoff control or mitigation of the urban heat island effect. However, new types of mobility and connections between built-up areas and GBI need to be balanced by a new institutional framing (what is allowed, what is promoted). The same goes for GBI itself and the different benefits it has to offer: institutional arrangements and officially recognized and sanctioned uses are under revision seeking to strike a new balance between conflicting interests. For example, Collserola Park authorities are restricting mass recreational activities, such as trail races, in order to minimize the impacts on biodiversity and habitat services. Participatory approaches to capture different societal perceptions and demands are already in place, although the alignment of new GBI, institutional regulations and diverse stakeholder perceptions and interests is one of the main challenges of Barcelona's GBI planning in the forthcoming years. For example, there has been a recent push for naturalization of GBI elements to increase their climate change resilience (e.g., turning irrigated grass lawns into Mediterranean meadows that dry out during the summer), done in dialogue with stakeholders. However, even if some people have embraced new management approaches better suited to local (and future) conditions with more frequent extreme climate events such as heat waves, others still prefer traditional practices and idealized views of nature.

In all, awareness of—and methods for comprehensively assessing and aligning—the three systemic filters will be necessary to provide a sustainable flow of GBI benefits over time in a more resilient city of Barcelona.

Change—or, rather, simultaneous processes of interlinked changes—needs to be accounted for in both studies and governance of ES benefit flows. As is evident from the account of different benefit flows and filter effects, building resilience around flows of multiple benefits cannot rely on just one approach, however holistic it may be. Working with and through the three filters can be complementary or contradictory, and they may have indirect consequences for GBI itself, as well as who gets to benefit from it. The approach outlined in this article provides new ground for asking questions and reevaluating existing strategies for building resilience. Strategies for managing and promoting multifunctionality, when the different ES are inherently different in how they are linked up to the three filters, need to take different justice dimensions into account. For example, strategies drawing on institutional factors raise questions of representative democracy, accountability, and legitimacy in governance (e.g., Cosens 2013). Furthermore, our approach emphasizes that distributional effects are not necessarily spatially and temporally immediate and that trade-offs between ES or the interests of different beneficiaries need to be addressed at larger temporal and spatial scales. Processes such as green gentrification (e.g., Anguelovski et al. 2018, Łaszkiewicz et al. 2018) serve as examples of how the implementation of new urban GBI can change the access to benefits by changing neighborhood demographic structures and in turn lead to unjust outputs in the mid- or long term, including the displacement of the most vulnerable. Targeted strategies, with their specific emphasis on different filters or subsets of ES, will always favor some actors and interests rather than others. Finding ways to balance different strategies and the scales at which this balance must be struck is at a premium, and this can only be done in a specific place at a specific time.

## Conclusions

With functioning ecosystems as the baseline for continued human well-being in the face of future changes, GBI will play an ever more important role in ensuring the resilience and sustainability of and within urban areas. However, the effectiveness of GBI in delivering its full potential of societal benefits is largely determined by the wider system in which the GBI is embedded. We suggest that careful consideration of three systemic filters will help GBI solutions to address environmental as well as social challenges. In short, the approach suggests that GBI functionality is a transdisciplinary and cross-sectoral issue and that the interactions and intersections between different factors are at least as decisive as the quality and accessibility of GBI itself. It also suggests that an improved understanding of the conditions under which different types of GBI components deliver multiple interconnected benefits and how these are received by diverse groups of beneficiaries will also enable upscaling of good examples and the effective implementation of different nature-based solution designs. There may also be trade-offs between the current desired state when it comes to a fair distribution of benefits and viable strategies, designs, or system configurations that could support continued flows of benefits across diverse futures.

Methodologically, we argue that there are no specific frameworks or standard methods that will work across all cases, especially not given the diverse nature of benefits and their interactions with the three filters; instead, we seek to identify which questions to ask and provide input to an informed discussion of the relevant knowledge needs for a specific case. The presented unifying systems approach and the logic behind can help as a generic approach to positioning and aligning different methods and data, as well as different theoretical components and themes. Especially, it can help us address new research questions or reevaluate old case studies and truths.

## Author Biographical

Erik Andersson (erik.andersson@su.se) and Timon McPhearson are affiliated with the Stockholm Resilience Centre, at Stockholm University, in Stockholm, Sweden. Erik Andersson is affiliated with the North-West University, in Potchefstroom, South Africa. Johannes Langemeyer and Francesc Baró are affiliated with the Institute of Environmental Science and Technology at the Universitat Autònoma de Barcelona, in Cerdanyola del Vallès, Spain. Johannes Langemeyer and Francesc Baró are affiliated with the Hospital del Mar Medical Research Institute, in Barcelona, Spain. Sara Borgström is affiliated with the Department of Sustainable Development, Environmental Science and Engineering, at the Royal Institute of Technology—KTH, in Stockholm, Sweden. Timon McPhearson is affiliated with the Urban Systems Lab, The New School, in New York, New York. Timon McPhearson is affiliated with the Cary Institute of Ecosystem Studies, in Millbrook, New York. Dagmar Haase is affiliated with the Institute of Geography at Humboldt Universität zu Berlin, in Berlin, Germany. Dagmar Haase is affiliated with the Helmholtz Centre for Environmental Research—UFZ, Department of Computational Landscape Ecology, in Leipzig, Germany. Jakub Kronenberg is affiliated with the Faculty of Economics and Sociology at the University of Lodz, in Lodz, Poland. David N. Barton is affiliated with the Norwegian Institute for Nature Research, in Trondheim, Norway. McKenna Davis, Sandra Naumann, and Lina Röschel are affiliated with the Ecologic Institute, in Berlin, Germany.
